# Construction Notes

**Published:** 1894-01-06

**Authors:** 


### CONSTRUCTION NOTES.

### Reflooring of the Women's Wards in Addenbrooke's Hospital, Cambridge.

When the hospital was undergoing alterations during the early part of the year it was found that the state of the floors left much to be desired, and that unless they were relaid with hard wood the sanitary condition of the wards would still be defective. A special effort was made to meet the expense, and a sum of over £300 was contributed to enable the governors to carry out the work. The governors contributed an equal sum from the general funds, and this sufficed to put in excellent condition two surgical and one medical ward. The improvement has been most marked, and it is now possible to keep these wards sweet and clean without the incessant damp and effluvia which accompany the scrubbing and scouring of rough boards. The refloored wards are, however, all on the men's side of the hospital, while two, one medical and one surgical, on the women's side are stil subject to the old disadvantages. In the hope that the liberality of the friends of the hospital is not yet exhausted, and in view of the great importance of putting the whole of the wards into the sound and healthful condition which is desirable in the interest of the suffering patients, the governors have opened an account at Messrs. Mortlock's, Cambridge, for the reflooring of Hatton and Victoria Wards. If by next summer the public contribute half the estimated expense (some £200) to this account, the governors are pepared to carry out this needed improvement during the ensuing Long Vacation. Any friend who is interested in the welfare of Addenbrooke's has only to visit the men's wards in their new condition, and compare them with the women's wards, to be convinced of the desirability of completing the work and rendering the hospital as a whole above reproach from a sanitary point of view.

